# Supplementary material for: Occupational Well-Being of Multidisciplinary PHC Teams: Barriers/Facilitators and Negotiations to Improve Working Conditions
Source: Int J Environ Res Public Health. 2022 Nov 29;19(23):15943. doi: 10.3390/ijerph192315943 (PMC9737019; doi:10.3390/ijerph192315943)
Supplement: Supplementary file 1 [file ijerph-19-15943-s001.zip › ijerph-1975072-supplementary.pdf]

Supplementary Materials (Figure S1, Figure S2, Table S1 and Table S2)

Figure S1. Detailed overview of the Inventory Well-Being at Work – (IWBW-13) *

INVENTORY WELL-BEING AT WORK – IBET 13

The following statements concern your current job at a Primary Health Care (PHC) service and the facility (Health Department) where you work.

PLEASE INDICATE AS HONESTLY AS POSSIBLE THE DEGREE TO WHICH YOU AGREE OR DISAGREE WITH EACH OF THE STATEMENTS.

Choose a score (from 1 to 5) that best represents your level of agreement or disagreement with each statement.

1. Completely disagree	2. Disagree	3. Nor agree, nor disagree	4. Agree	5. Completely agree

( ) 1. I am content with the company I work for.
( ) 2. I am enthusiastic about the company I work for.
( ) 3. The hours I spend at work are the best hours of my day.
( ) 4. I am interested in the company I work for.
( ) 5. I am satisfied with my work compared to my efforts at work.
( ) 6. I am excited about the company I work for.
( ) 7. The greatest satisfactions in my life come from my work.
( ) 8. I am satisfied with the level of interest my tasks arouse in me.
( ) 9. The most important things that happen in my life involve my work.
( ) 10. I am satisfied with the understanding reached between my boss and me.
( ) 11. I am proud of the company I work for.
( ) 12. I eat, live, and breathe my work.
( ) 13. I am satisfied with the promotion opportunities provided by this company.

Figure S1. Adapted from: New Measures of Organizational Behavior (electronic resource): Diagnostic and Management Tools. [Novas Medidas do Comportamento Organizacional (recurso eletrônico): Ferramentas de Diagnóstico e de Gestão] Organizer: Mirlene Maria M. Siqueira. – Electronic Data. Porto Alegre: Artmed, 2014. *Own translations. This does not constitute a validated scale in English and is for informational purposes only.

Figure S2. Individual Questionnaire applied to Primary Health Care workers – *Questions selected for a study on well-being at work: sociodemographic and occupational characterization; ICF occupational functionality; perception of occupational risks; negotiation for improving working conditions and reasons for negotiation. *Own translations. These do not constitute a validated questionnaire in English and are for informational purposes only. (following).

Individual Questionnaire —Selected Questions

Individual Questionnaire applied to Primary Health Care workers – Questions selected for a study on well-being at work: sociodemographic and occupational characterization; ICF occupational functionality; perception of occupational risks; negotiation for improving working conditions and reasons for negotiation.

DATA COLLECTION INSTRUMENT – Primary Health Care (PHC)

INTERVIEW Nº: ______________
TEAM: ______________
PROFESSION: ______________
DATE OF INTERVIEW: ______________
START: _________ hours
FINISH: _________ hours

INTERVIEWER: ____

PART I – PRIMARY HEALTH CARE (PHC) SERVICE CHARACTERIZATION

01. Basic health units (BHU)

0. BHU – Regular
1. Family BHU
2. NASF (Family Health Support Center)
3. BHU – 24h

02. City

0. Rio Grande (Please specify BHU): ____________________________________
1. São José do Norte (Please specify BHU): ____________________________________

PART II – INTERVIEWEE CHARACTERIZATION: BHU WORKER

03. What is your age? ____________ years old

Place of birth: ____

04. What is your race?

0. Caucasian
1. Afro-descendant
2. Asian
3. Mixed race
4. Indigenous

05. What is your sex?

0. Female
1. Male

06. What is your weight? _______________kg

07. What is your height? _______________meter

08. What is your educational level?

0. Middle school
1. Incomplete high school
2. Complete high school
3. Vocational School
4. Some undergraduate studies
5. Bachelor's degree
6. Technician (Please specify): ____
7. Specialization (Please specify): ____
8. Master's degree
9. Doctoral degree

09. What is your profession?

0. Nurse
1. Physician
2. Nursing technician
3. Community health agent
4. Dentist
5. Oral health technician/Assistant
6. Other (Please specify): ______________________________________

10. How long have you been in this profession? _________years and _________months

11. How long have you worked in a PHC service? _________years and _________months

12. How many hours/week do you work in the PHC service? __________hours

13. What is your work schedule in the PHC service?

0. Daytime
1. Nighttime
2. Nighttime/Daytime, depending on the day schedule
3. Other (Please specify): ___________________________________

14. Do you have a second job?

0. No
1. Yes (Please specify): ___________________________________

15. What is your total weekly workload? _____________hours

16. What is your marital status?

0. Single
1. Married/Consensual union
2. Separate/Divorced
3. Widowed

17. **Do you have children?**

0. No
1. Yes (How many?): ____________ children

18. **How many people live in your household, including you?** ____________peoples

19. **What is your monthly income?** (Based on the minimum wage (MW) of 2020 of R$ 1,039.00)

0. Up to 2 times the MW
1. Between 2 and 4 times the MW
2. Between 4 and 6 times the MW
3. Between 6 and 8 times the MW
4. Between 8 and 10 times the MW
5. More than 10 times the MW

PART III – CHARACTERIZATION: HEALTH/WORK/ENVIRONMENT

(Questions addressing occupational functionality – ICF)

---

20. **What is your level of difficulty in solving problems, considering your current health state?**

0. No difficult
1. Mild difficulty
2. Moderate difficult
3. Severe difficulty
4. Complete difficulty

21. **What is your level of difficulty in solving problems, considering your current environment?**

0. No difficult
1. Mild difficulty
2. Moderate difficult
3. Severe difficulty
4. Complete difficulty

22. **What is your level of difficulty in performing multiple tasks, considering your current health state?**

0. No difficult
1. Mild difficulty
2. Moderate difficult
3. Severe difficulty
4. Complete difficulty

23. **What is your level of difficulty in performing multiple tasks, considering your current environment?**

0. No difficult
1. Mild difficulty
2. Moderate difficult
3. Severe difficulty
4. Complete difficulty

24. **What is your level of difficulty in performing work routines within PHC, considering your health state?**

0. No difficult
1. Mild difficulty
2. Moderate difficult
3. Severe difficulty
4. Complete difficulty

25. **What is your level of difficulty in performing work routines within PHC, considering your work?**
0. No difficult
1. Mild difficulty
2. Moderate difficult
3. Severe difficulty
4. Complete difficulty

26. **Do you believe community members' general/specific opinions or beliefs are barriers or facilitators influencing your well-being or individual actions? Please rate the degree of such barriers/facilitators:**

Barriers	Facilitators
( ) 0 NO Barriers	( ) 0 NO Facilitator
( ) 1 MILD Barriers	( ) +1 MILD Facilitator
( ) 2 MODERATE Barriers	( ) +2 MODERATE Facilitator
( ) 3 SEVERE Barriers	( ) +3 SUBSTANTIAL Facilitator
( ) 4 COMPLETE Barriers	( ) +4 COMPLETE Facilitator

27. **Do you believe your co-workers' individual attitudes, general/specific opinions or beliefs are barriers or facilitators influencing your well-being or individual actions? Please rate the degree of such barriers/facilitators:**

Barriers	Facilitators
( ) 0 NO Barriers	( ) 0 NO Facilitator
( ) 1 MILD Barriers	( ) +1 MILD Facilitator
( ) 2 MODERATE Barriers	( ) +2 MODERATE Facilitator
( ) 3 SEVERE Barriers	( ) +3 SUBSTANTIAL Facilitator
( ) 4 COMPLETE Barriers	( ) +4 COMPLETE Facilitator

PART IV – CHARACTERIZATION: OCCUPATIONAL RISK PERCEPTION

28. **Do you identify <u>physical</u> occupational risks in your workday at the PHC service?** (Physical risks are forms of energy to which workers are exposed, such as noise, vibration, heat, cold, humidity, ionizing and non-ionizing radiation, pressure, and others).
1. Yes
2. No

29. **Do you identify <u>chemical</u> occupational risks in your workday at the PHC service?** (Chemical risks are substances, compounds, or products that can enter an individual's body through the respiratory route, including gases, dust, fumes, or vapors, in addition to substances that can be absorbed by the body through the skin or by ingestion).
1. Yes
2. No

30. **Do you identify <u>biological</u> occupational risks in your workday at the PHC service?** (Biological risks are bacteria, viruses, fungi, and protozoa; preventive measures depend on the pathogenicity to which workers are exposed).
1. Yes
2. No

31. **Do you identify <u>ergonomic</u> occupational risks in your workday at the PHC service?** (Ergonomic risks are related to work situations that are not appropriate for workers)
1. Yes
2. No

32. **Do you identify psychosocial occupational risks in your workday at the PHC service?** (Psychosocial risks are factors that can change and harm workers' health)
    1. Yes
    2. No

PART V – CHARACTERIZATION: NEGOTIATIONS INTENDED TO IMPROVE WORKING CONDITIONS

---

33. **With "Whom," do you negotiate to improve PHC working conditions? (MULTIPLE CHOICE)**
    ( ) Workers/Co-workers
    ( ) Workers union
    ( ) Managers/supervisors
    ( ) Community members
    ( ) Occupational safety workers

34. **Please list the reasons you negotiate improved health and safe working conditions at the PHC service: (MULTIPLE CHOICE)**

    ( ) To change the conditions of exposure to **physical** occupational risks
    ( ) To change the conditions of exposure to **chemical** occupational risks
    ( ) To change the conditions of exposure to **biological** occupational risks
    ( ) To change the conditions of exposure to **ergonomic** occupational risks
    ( ) To change the conditions of exposure to occupational **accidents**
    ( ) To change the conditions of exposure to **psychosocial** occupational risks
    ( ) To arrange the acquisition of PPE.
    ( ) To encourage employee compliance with PPE.
    ( ) Salaries
    ( ) To discuss/change work goals (productivity)
    ( ) To resolve conflicts of interest (concerning working dynamics, days off, payment and/or sick leave).
    ( ) Promote educational qualification
    ( ) To redistribute working shifts
    ( ) To obtain the right to health (healthcare insurance/plan – public or private)
    ( ) Workload (breaks, intervals)
    ( ) To improve interaction/communication among workers
    ( ) To improve interaction/communication between workers and employers
    ( ) Resources provided to labor unions

Observation:
This set of questions was selected from a more extensive questionnaire to develop this paper.

Supplementary Materials (Table S1 and Table S2)

Table S1 and S2 present the complete Multivariate Linear Regression model for the Inventory Well-Being at Work scores.

Table S1. Multivariate Linear Regression Analysis to assess factors independently associated with the Commitment and Satisfaction dimension of the Inventory Well-being at Work.

Variables	b (95%CI)	Beta	p-value
**Age (years)**	0.00 (-0.01 a 0.01)	0.038	0.550
**Sex** Male	0.16 (-0.06 a 0.38)	0.076	0.155
**Marital Status**			
Single	0.15 (-0.10 a 0.39)	0.092	0.239
Married/Consensual union	0.14 (-0.08 a 0.37)	0.099	0.205
Separated/Divorced	0.00	-	-
Widowed	0.43 (-0.10 a 0.96)	0.075	0.110
**City Rio Grande**	0.26 (0.08 a 0.44)	0.138	0.005
**Profession**			
Nurse	0.31 (0.11 a 0.51)	0.152	0.003
Physician	-0.23 (-0.44 a -0.01)	-0.105	0.040
Nursing technician/assistant	0.27 (0.10 a 0.45)	0.156	0.002
Community health agent	0.00		
Dentist	0.42 (0.08 a 0.76)	0.117	0.015
Oral health technician/assistant	0.12 (-0.24 a 0.47)	0.032	0.519
Other	0.12 (-0.40 a 0.64)	0.022	0.656
**Years working at the PHC service**	-0.01 (-0.02 a -0.00)	-0.107	0.027
**Weekly hours**	-0.00 (-0.02 a 0.01)	-0.027	0.590
**Physical Occupational Risk**	-0.32 (-0.56 a -0.08)	-0.132	0.010
**Chemical Occupational Risk**	-0.21 (-0.39 a -0.03)	-0.118	0.021
**Biological Occupational Risk**	0.10 (-0.14 a 0.35)	0.048	0.401
**Ergonomic Occupational Risk**	0.05 (-0.33 a 0.43)	0.013	0.809
**Health Status: Level of difficulty in solving problems**	0.02 (-0.10 a 0.15)	0.025	0.744
**Current environment: Level of difficulty in solving problems**	-0.15 (-0.23 a -0.07)	-0.197	<0.001
**Health state: Level of difficulty in performing multiple tasks**	0.05 (-0.07 a 0.16)	0.058	0.456
**Current environment: Level of difficulty in performing multiple tasks**	-0.08 (-0.19 a 0.03)	-0.110	0.167
**Health state: Level of difficulty in performing work routines**	-0.00 (-0.14 a 0.13)	-0.01	0.954
**Current environment: Level of difficulty in performing work routines**	-0.10 (-0.23 a 0.03)	-0.129	0.133
**Scores assigned to the community's attitudes: Barriers**	-0.02 (-0.10 a 0.06)	-0.031	0.575
**Scores assigned to the colleagues' attitudes: Barriers**	-0.09 (-0.16 a -0.02)	-0.124	0.017
**Negotiates to improve the PHC working conditions w/ workers/colleagues**	0.00 (-0.16 a 0.17)	0.002	0.979

**Negotiates to improve the PHC working conditions w/ labor union**	0.03 (-0.22 a 0.27)	0.011	0.841
**Negotiates to improve the PHC working conditions w/ managers/supervisors**	0.44 (0.31 a 0.58)	0.308	<0.001
**Negotiates to improve the PHC working conditions w/ community members**	0.40 (0.17 a 0.63)	0.168	0.001

Table S1. Multivariate Linear Regression Analysis to assess factors independently associated with the Commitment and Satisfaction dimension of the Inventory Well-being at Work. (continuation)

**Variables**	**b (95%CI)**	**Beta**	**p-value**
**Negotiates to improve the PHC working conditions w/ occupational safety workers**	0.11 (-0.32 a 0.53)	0.025	0.628
**Reasons to negotiate: Change the conditions of exposure to ergonomic risks**	-0.07 (-0.26 a 0.11)	-0.048	0.444
**Reasons to negotiate: Change the conditions of exposure to occupational accidents**	0.02 (-0.18 a 0.22)	0.012	0.840
**Reasons to negotiate: Change the conditions of exposure to psychosocial risks**	-0.10 (-0.27 a 0.07)	-0.066	0.264
**Reasons to negotiate: Arrange the acquisition of PPEs**	0.07 (-0.09 a 0.22)	0.048	0.381
**Reasons to negotiate: Salaries**	-0.05 (-0.21 a 0.10)	-0.036	0.494
**Reasons to negotiate: Discuss/change work goals**	0.07 (-0.09 a 0.23)	0.047	0.401
**Reasons to negotiate: Resolve conflicts of interest**	-0.11 (-0.28 a 0.06)	-0.076	0.209
**Reasons to negotiate: Promote educational qualification**	0.03 (-0.13 a 0.19)	0.023	0.687
**Reasons to negotiate: Redistribute working shifts**	-0.23 (-0.44 a -0.02)	-0.104	0.032
**Reasons to negotiate: Obtain the right to health at work**	-0.10 (-0.26 a 0.08)	-0.063	0.274
**Reasons to negotiate: Working hours**	-0.04 (-0.22 a 0.14)	-0.026	0.648
**Reasons to negotiate: Discuss the resources provided to labor unions**	0.01 (-0.27 a 0.28)	0.003	0.950

Table S2. Multivariate Linear Regression Analysis to assess factors independently associated with the Job Involvement dimension of the Inventory Well-being at Work.

Variables	b (95%CI)	Beta	p-value
**Age (years)**	0.19 (-0.06 a 0.45)	0.090	0.138
**Sex Male**			
Marital Status	0.23 (-0.03 a 0.48)	0.139	0.082
Single	0.21 (-0.02 a 0.44)	0.140	0.076
Married/Consensual union	0.00		
Separated/Divorced	0.47 (-0.15 a 1.08)	0.082	0.136
**City Rio Grande**	0.21 (-0.01 a 0.43)	0.111	0.064
**Second job**	0.17 (-0.04 a 0.38)	0.094	0.106
**Profession**			
Nurse	0.21 (-0.01 a 0.42)	0.099	0.057
Physician	0.04 (-0.20 a 0.28)	0.018	0.744
Nursing technician/assistant	0.46 (0.28 a 0.64)	0.260	<0.001
Community health agent	0.00		
Dentist	0.33 (-0.04 a 0.69)	0.089	0.080
Oral health technician/assistant	0.06 (-0.31 a 0.43)	0.017	0.745
Other	-0.08 (-0.62 a 0.47)	-0.014	0.785
**Years working at the PHC service**	-0.01 (-0.02 a 0.01)	-0.050	0.412
**Work shift**			
Daytime	0.45 (-0.13 a 1.02)	0.188	0.127
Nighttime	0.77 (0.03 a 1.52)	0.156	0.042
Nighttime/Daytime	0.71 (0.08 a 1.34)	0.243	0.028
Other	0.00		
**Physical Occupational Risk**	-0.05 (-0.34 a 0.25)	-0.019	0.755
**Chemical Occupational Risk**	-0.32 (-0.51 a -0.14)	-0.176	0.001
**Biological Occupational Risk**	-0.25 (-0.63 a 0.13)	-0.082	0.193
**Ergonomic Occupational Risk**	0.19 (-0.08 a 0.47)	0.088	0.160
**Psychosocial Occupational Risk**	-0.31 (-0.73 a 0.11)	-0.080	0.151
**Health Status: Level of difficulty in solving problems**	0.09 (-0.05 a 0.22)	0.106	0.199
**Current environment: Level of difficulty in solving problems**	-0.08 (-0.20 a 0.05)	-0.098	0.216
**Health Status: Level of difficulty in performing multiple tasks**	0.06 (-0.07 a 0.19)	0.076	0.370
**Current environment: Level of difficulty in performing multiple tasks**	-0.07 (-0.19 a 0.06)	-0.088	0.316
**Health Status: Level of difficulty in performing working routines**	-0.04 (-0.19 a 0.11)	-0.048	0.620
**Current environment: Level of difficulty in performing working routines**	-0.17 (-0.25 a -0.09)	-0.218	<0.001
**Scores assigned to the community's attitudes: Barriers**	0.00 (-0.09 a 0.09)	0.000	0.995
**Scores assigned to the community's attitudes: Facilitators**	0.04 (-0.03 a 0.11)	0.063	0.254
**Scores assigned to the colleagues' attitudes: Barriers**	0.02 (-0.06 a 0.10)	0.027	0.649

Table S2 – Multivariate Linear Regression Analysis to assess factors independently associated with the Job Involvement dimension of the Inventory Well-being at Work. (continuation)

Variables	b (95%CI)	Beta	p-value
**Negotiates to improve the PHC working conditions w/ workers/colleagues**	0.08 (-0.10 a 0.26)	0.055	0.376
**Negotiates to improve the PHC working conditions w/ labor union**	-0.23 (-0.49 a 0.04)	-0.095	0.089
**Negotiates to improve the PHC working conditions w/ managers/supervisors**	0.39 (0.25 a 0.54)	0.272	<0.001
**Negotiates to improve the PHC working conditions w/ occupational safety workers**	0.40 (-0.05 a 0.85)	0.095	0.079
**Reasons to negotiate: Change exposure to occupational accidents**	-0.12 (-0.32 a 0.08)	-0.071	0.240
**Reasons to negotiate: Change exposure to psychosocial risk**	-0.03 (-0.22 a 0.15)	-0.022	0.724
**Reasons to negotiate: Discuss/change work goals**	0.01 (-0.16 a 0.19)	0.009	0.887
**Reasons to negotiate: Resolve conflicts of interest**	-0.05 (-0.23 a 0.13)	-0.033	0.601
**Reasons to negotiate: Redistribute working shifts**	-0.25 (-0.48 a -0.03)	-0.111	0.030
